# Statistical evaluation of the medium components for the production of high biomass, α-amylase and protease enzymes by *Piriformospora indica* using Plackett–Burman experimental design

**DOI:** 10.1007/s13205-013-0168-7

**Published:** 2013-09-06

**Authors:** S. Swetha, Ajit Varma, T. Padmavathi

**Affiliations:** 1 Department of Microbiology, Centre of PG Studies, Jain University, 9th Main, Jayanagar 3rd Block, Bangalore, India; 2 Amity Institute of Herbal and Microbial Studies, Sector 125, Noida, 201303 UP India

**Keywords:** *Piriformospora indica*, Submerged fermentation, Plackett–Burman experimental design, α-Amylase, Protease

## Abstract

*Piriformospora indica*, a member of basidiomycota is an axenically cultivable endophytic fungus which exerts plant growth promoting effects on its host plant. *P. indica* is known to produce α-amylase and protease. Since the organism exhibits beneficial role in plant growth promotion, achieving high biomass is immensely essential. Hence to enable the commercial production, screening of medium components is a necessary step. The present paper investigates the screening of medium components using Plackett–Burman experimental design wherein the parameters such as α-amylase, protease and biomass have been examined. The parameters α-amylase, protease and biomass was found to vary from 0.25 to 0.45 mg^−1 ^ml^−1 ^min^−1^, 0.1 to 0.15 mg^−1 ^ml^−1^ h^−1^ and 0.8 to 22.6 g l^−1^, respectively, in 16 runs which demonstrates the strong influence of the medium components.

## Introduction

*Piriformospora indica* is an axenically cultivable, facultative root endophytic fungus belonging to basidiomycota possessing simple dolipore septum with continuous and straight parenthosomes. It was isolated from the rhizosphere of the woody shrubs *Prosopsis juliflora* and *Zizyphus nummularia* growing in Indian Thar desert (Varma et al. 1998). *P. indica* enhances the growth and yield of plants, protects against biotic (resistance against pathogens) and abiotic stress (salt stress) (Rai et al. 2001, Waller et al. 2008) by colonizing a broad spectrum of mono and dicotyledenous plants. It also produces a large number of thick walled, pear shaped chlamydospores having longer shelf life. The sporulating nature of this fungus has made it competent enough for exploiting it for commercial application (Kumar et al. 2011).

Microbial enzymes find increasing industrial applications; among them amylases and proteases occupy large share of the total enzyme market. α-Amylases and proteases have wide applications in starch processing, brewing, alcohol production, dairy industries, and textile industries, pharmaceutical and detergent industries, respectively (Sumrin et al. 2011; Saxena and Singh 2010).

The cell growth, spore production and accumulation of metabolic products are strongly influenced by the medium components such as carbon sources, nitrogen sources, various inorganic salts and trace elements. Screening of medium components and their optimization is therefore an important criterion for large-scale production. Classical method, factorial combination of medium optimization involving one variable at a time by keeping others at fixed level fails as it is laborious, time consuming; moreover, it does not guarantee the optimal conditions. Hence statistical approach such as Plackett–Burman design (provide statistical model) is a useful tool for the screening of nutrients as it helps in determining the significant impact on growth rate, which in turn aids in understanding the interactions among the process parameters at different levels (Rajendran et al. 2007). The use of statistical experimental design in medium optimization has gained considerable attention in recent years and also number of publications describing the application of these methods for the production of various enzymes and biomolecules has appeared in the literature (Seraman et al. 2010). However, there seems to be no reports available for the production of α-amylases and proteases by *P. indica*. The present paper demonstrates the screening of the important medium components affecting the large-scale cultivation of *P. indica* and also the production of α-amylase and protease enzymes by *P. indica*, which has tremendous applications in the field of plant biotechnology as a biological hardening tool, biofertilizer and biocontrol agent.

## Materials and methods

### Microorganism and culture maintenance

The culture of *P. indica* with an accession number of AF014929 USA was obtained from Prof. Ajit Varma (Amity Institute of Herbal and Microbial Studies, Noida, India). The stock culture was maintained on potato dextrose agar at optimum conditions and stored at 4 °C for further studies.

### Screening of medium components

A total of 12 variables including different carbon, nitrogen sources, trace element solution [zinc sulphate (ZnSO_4_·7H_2_O), boric acid (H_3_BO_3_), manganous chloride (MnCl_2_), cobaltous chloride (CoCl_2_), copper sulphate (CuSO_4_) and ammonium molybdate ((NH_4_)_6_Mo_7_O_24_·4H_2_O) and 20× salt solution [sodium nitrate (NaNO_3_), potassium chloride, magnesium sulphate (MgSO_4_·7H_2_O) and potassium di hydrogen phosphate (KH_2_PO_4_)] at different levels as per the statistical model were used along with potato dextrose media for screening (Table 1).

**Table 1:** Plackett–Burman experimental design for the evaluation of 12 variables for α-amylase, protease and biomass

Run	Soluble starch	Maltose	Sucrose	Glycerol	Carboxy methyl cellulose	Sodium nitrate	Ammonium chloride	Malt extract	Peptone	Yeast extract	TE solution	20× salt solution
1	+	+	−	+	−	+	+	−	+	−	+	+
2	+	+	+	−	+	+	−	+	−	−	+	−
3	+	−	+	−	−	+	+	−	+	+	−	−
4	+	+	−	+	−	−	−	+	+	−	−	−
5	+	−	−	−	−	+	+	+	−	−	+	+
6	+	+	+	−	+	−	+	−	−	−	−	+
7	−	+	−	−	+	+	−	−	+	+	−	+
8	+	−	−	+	+	+	−	+	−	+	−	+
9	+	−	−	+	+	−	+	−	−	+	+	−
10	−	+	+	+	−	−	−	−	−	+	+	+
11	−	−	+	+	+	+	−	−	+	−	+	−
12	+	−	+	−	−	−	−	+	+	+	+	+
13	−	−	+	+	+	−	+	+	+	−	−	+
14	−	+	−	−	+	−	+	+	+	+	+	−
15	−	+	+	+	−	+	+	+	−	+	−	−
16	−	−	−	−	−	−	−	−	−	−	−	−

+ higher level (0.1 g), − lower level (0.05 g)

### Submerged fermentation

1 × 10^−6^ ml^−1^ of the culture was inoculated to all the flasks (16 runs) designed as per the statistical experimental design and these flasks were incubated at 30 °C under continuous shaking conditions (120 rpm) and the response was measured in terms of biomass, amylase and protease production.

### Measurement of cell growth

The culture broth was filtered through pre-weighed Whatman No. 1 filter paper, dried in a hot air oven at 60 °C for 48–72 h and the growth of *P. indica* was expressed in terms of dry cell weight per liter of the culture broth.

### α-Amylase activity

α-Amylase activity was performed using 1 % soluble starch as substrate (optimum condition) followed by the estimation of reducing sugars using dinitrosalicylic acid (Miller 1959). One unit of amylase activity was defined as the amount of enzyme that liberates reducing sugar equivalent to 1.0 mg glucose under specific assay conditions.

### Protease activity

Protease activity using casein as substrate was performed according to Rodarte et al. (2011) with some modifications. The reaction mixture containing cell-free extract, 0.5 % casein along with citrate buffer of pH 5 was incubated for 4 h at room temperature followed by the addition of 5 % trichloroacetic acid and centrifuged at 8,000 rpm for 10 min. The supernatant was further analyzed by Lowry’s method (Lowry et al 1951). One unit of the protease activity was defined as the amount of enzyme required to liberate 1 μg of tyrosine per hour under the experimental conditions.

### Plackett–Burman design

The Plackett–Burman design based on the first order model was used to screen and evaluate the important medium components that influence the production of α-amylase and protease. All the experiments were carried out according to designed matrix using the equation $Y=\;\beta_{o}+\sum \beta_{i}X_{i}\left(i=\;l\ldots k\right)$, where *Y* is the estimated target function (yield of α-amylase/protease/biomass), *β*_*o*_ is a model intercept, *β*_*i*_ is the regression coefficient. *X* is the independent variable and *k* is the number of variables (Rajendran et al. 2007; Seraman et al. 2010). The Student’s *t* test was performed to determine the significance of each variable employed. The regression coefficient was determined by least square method.

## Results

The coefficient and the *p* values of the factors were studied using the statistical software MATLAB. α-Amylase activity, protease activity and biomass were found to vary from 0.2555 to 0.4554 mg^−1 ^ml^−1 ^min^−1^, 0.1 to 0.158 mg^−1 ^ml^−1^ h^−1^ and 0.8 to 22.6 g l^−1^, respectively, in 16 runs which shows the strong influence of the medium components. The variables were classified into positive and negative variables based on their main effect which in turn highlights the direction of optimization. More than half of the considered parameters were significantly acting on the system. The components such as starch, glycerol, maltose, TE solution and salt solution have positive effect, whereas sodium nitrate and ammonium chloride has a negative effect on the above-mentioned activities.

**Fig. 1:**
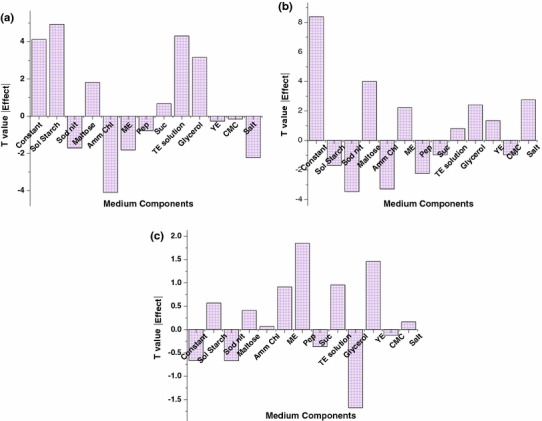
Pareto plots depicting the main effect of the variables on **a** α-amylase activity, **b** protease activity and **c** biomass

Pareto plots give a clear view about the role of each variable (Fig. 1a–c). Soluble starch, maltose, sucrose, TE solution and glycerol had a positive effect on α-amylase activity where as sodium nitrate, ammonium chloride, yeast extract, malt extract, carboxymethyl cellulose and salt solution had negative effect as shown in Fig. 1a. Figure 1b depicts the pareto plot of protease activity which reveals that maltose, malt extract, TE solution, glycerol, yeast extract and salt solution had a positive effect, whereas the soluble starch, sodium nitrate, ammonium chloride, peptone, sucrose, carboxymethyl cellulose had a negative effect. Soluble starch, maltose, ammonium chloride, malt extract, peptone, TE solution, yeast extract and salt solution supported in enhancing the biomass but sodium nitrate, sucrose, glycerol and carboxymethyl cellulose depicted negative role in the enhancement of growth of *P. indica* (Fig. 1c).

Total terms in the quadratic equation were 91 including 1 constant, 66 interactions, 12 linear and 12 pure quadratic terms.

To obtain the relevant parameter, the constant term and the linear effect alone were taken into consideration, and the following formula was used.

$$\begin{array}{cc} & C1+\;C2\times \text{soluble starch}+\;C3\times \text{sodium nitrate}+\;C4\times \text{maltose}+\;C5\times \text{ammonium chloride}\\ & \;+\;C6\times \text{malt extract}+C7\times \text{peptone}+C8\times \text{sucrose}+C9\times \text{TE solution}+\;C10\times \text{glycerol}\\ & \;+\;C11\times \text{yeast extract}+C12\times \text{CMC}+C13\times \text{salt solution}. \end{array}$$

### α-Amylase activity

**Fig. 2:**
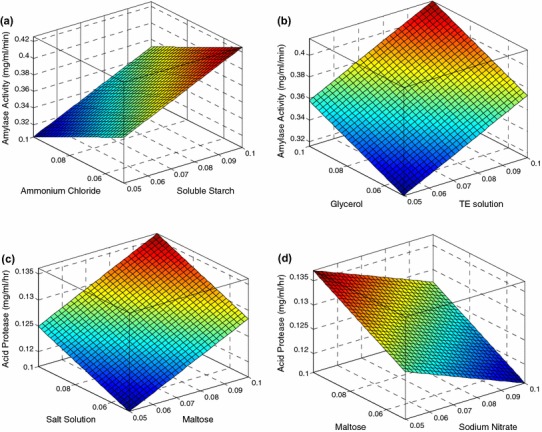
3D response plots showing the effects of independent variables on α-amylase and protease activity

The linear effect of soluble starch, ammonium chloride and TE solution were found to be more significant than other variables for the production of α-amylase enzyme by *P. indica* as given in Table 2. Figure 2 represents the response surface plots of the effect of various combinations of independent variables such as (1) ammonium chloride and soluble starch, (2) glycerol and TE solution for the production of α-amylase. The interactions of ammonium chloride and soluble starch on α-amylase production was significant as shown in Fig. 2a. Activity increased with increase in concentration of soluble starch, whereas the effect was opposite for ammonium chloride as depicted in Table 2. The maximum value observed was 0.43 mg^−1 ^ml^−1 ^min^−1^ at 0.05 % (w/v) of ammonium chloride and 0.1 % (w/v) of soluble starch. Similarly in case of glycerol and TE solution, the maximum value observed was 0.42 mg^−1 ^ml^−1 ^min^−1^ at 0.1 % (w/v) of glycerol and TE solution as the effect of both the variables were positive and dominating (Fig. 2b).

**Table 2:** Regression coefficient results from the data of central composite designed experiments for α-amylase activity and protease activity

Sl. no.	Components	α-Amylase activity		Protease activity	
		*t* stat	*p* value	*t* stat	*p* value
1	Constant	4.1280	0.0258	8.3769	0.0036
2	Soluble starch	4.9329	0.0160	−1.6904	0.1895
3	Sodium nitrate	−1.7097	0.1859	−3.4698	0.0404
4	Maltose	1.8143	0.1673	4.0036	0.0279
5	Ammonium chloride	−4.1014	0.0262	−3.2919	0.0460
6	Malt extract	−1.8143	0.1673	2.2242	0.1126
7	Peptone	−0.7736	0.4956	−2.2242	0.1126
8	Sucrose	0.6745	0.5483	−0.9787	0.3999
9	TE solution	4.3088	0.0230	0.8007	0.4818
10	Glycerol	3.1596	0.0509	2.4022	0.0957
11	Yeast extract	−0.2635	0.8093	1.3345	0.2743
12	CMC	−0.1532	0.8880	−0.9787	0.3999
13	Salt solution	−2.2273	0.1123	2.7581	0.0703

*p* value <0.05 is significant

### Protease activity

The effect of sodium nitrate, maltose and 20× salt solution were found to be more significant than those of other variables for the production of protease enzyme is as evident from the Table 2. Figure 2c, d represents the response surface plots of the effect of combinations of independent variables such as (1) maltose and sodium nitrate (2) salt solution and maltose for the production of protease enzyme. The interactions of maltose and sodium nitrate on protease production were significant as shown in Fig. 2c. With the increase in concentration of maltose, the activity increased where as the activity decreased with increase in concentration of sodium nitrate. The maximum value observed was 0.136 mg^−1 ^ml^−1^ h^−1^ at 0.1 % (w/v) of maltose and 0.05 % (w/v) of sodium nitrate. The interactions of salt solution and maltose gave the highest activity of 0.135 mg^−1 ^ml^−1^ h^−1^ at 0.1 % (w/v) of salt solution and 0.1 % (w/v) of maltose (Fig. 2d).

### Biomass

The linear effects of peptone, glycerol and yeast extract for biomass were found to be moderately significant. The biomass varied significantly with the concentration of glycerol, peptone and achieved the maximum biomass of 12 g l^−1^ at 0.1 % (w/v) of peptone and 0.05 % (w/v) of glycerol.

## Discussion

Kumar et al. (2012) have reported that the increase in concentration of soluble starch increased the production of α-amylase by *P. indica*. Starch is a preferable carbon source over glucose and its concentration has a direct effect on α-amylase production as reported by Gangadharan et al. (2006) and Tanyildizi et al. (2006). Babu and Satyanarayana (1993) and Narang and Satyanarayana (2001) reported that the organic nitrogen sources produce high α-amylase yield compared to inorganic nitrogen sources. This is in accordance with our results that describe positive impact of soluble starch and negative impact of inorganic nitrogen sources on α-amylase activity.

Carbon sources such as glucose, sucrose, maltose, etc., have a positive influence on the production of protease and also salts such as KH_2_PO_4_, MgSO_4_·7H_2_O and KCl have positive impact on protease production as reported by Kezia et al. 2011. Present study is in concordance with Luciana and Sato (2009) who reported that concentration of KH_2_PO_4_ plays an important role in production of proteolytic enzymes.

Carbon sources play a major role in growth of the microorganisms and also in obtaining high biomass. Present findings reveal that glycerol as a carbon source (economical source) and peptone as a nitrogen source promotes the cell growth and also yield, higher biomass which are in accordance with the earlier reports by Meinicke et al. (2012); Sreekumar and Soundarajan (2010).

The novelty of present paper lies in the screening of medium components to obtain higher enzyme activity and biomass of *P. indica*, which has tremendous applications in the field of plant biotechnology as a biological hardening tool, biofertilizer and biocontrol agent.

## Conclusion

The Plackett–Burman design based on the first order model to screen and evaluate the important medium components greatly influenced the production of α-amylase, protease and also biomass. α-Amylase, protease and biomass were found to vary from 0.25 to 0.45 mg^−1 ^ml^−1 ^min^−1^, 0.1 to 0.15 mg^−1 ^ml^−1^ h^−1^ and 0.8 to 22.6 g l^−1^, respectively, in 16 runs which showed the strong influence of the medium components. Soluble starch, ammonium chloride, TE solution and glycerol were found to be significant for α-amylase activity. Sodium nitrate, ammonium chloride, maltose and salt solution were found to be significant for protease activity. Peptone and glycerol supported well the production of high biomass.
